# The Association between Rapid Weight Loss and Body Composition in Elite Combat Sports Athletes

**DOI:** 10.3390/healthcare10040665

**Published:** 2022-04-01

**Authors:** Marius Baranauskas, Ingrida Kupčiūnaitė, Rimantas Stukas

**Affiliations:** 1 Faculty of Biomedical Sciences, Panevėžys University of Applied Sciences, 35200 Panevėžys, Lithuania; ingrida.kupciunaite@panko.lt; 2 Department of Public Health, Institute of Health Sciences, Faculty of Medicine, Vilnius University, 01513 Vilnius, Lithuania; rimantas.stukas@mf.vu.lt

**Keywords:** combat sports, elite athletes, rapid weight loss, extreme weight loss, weight management

## Abstract

Rapid Weight Loss (RWL) is a rapid reduction in weight over a short period of time seeking to attain the norm required for a competition in a particular weight category. RWL has a negative health impact on athletes including the significant muscle damage induced by RWL. This study aimed to identify the association between RWL and body composition among competitive combat athletes (*n* = 43) in Lithuania. Our focus was laid on the disclosure of their RWL practice by using a previously standardized RWL Questionnaire. The body composition of the athletes was measured by means of the standing-posture 8-12-electrode multi-frequency bioelectrical impedance analysis (BIA) and the electrical signals of 5, 50, 250, 550 and 1000 kHz. This non-experimental cross-sectional study resulted in preliminary findings on the prevalence and profile of RWL among combat athletes in Lithuania. 88% of the athletes surveyed in our study had lost weight in order to compete, with the average weight loss of 4.6 ± 2% of the habitual body mass. The athletes started to resort to weight cycling as early as 9 years old, with a mean age of 12.8 ± 2.1 years. The combination of practiced weight loss techniques such as skipping meals (adjusted Odd Ratio (AOR) 6.3; 95% CI: 1.3–31.8), restricting fluids (AOR 5.5; 95% CI: 1.0–31.8), increased exercise (AOR 3.6; 95% CI: 1.0–12.5), training with rubber/plastic suits (AOR 3.2; 95% CI: 0.9–11.3) predicted the risk of RWL aggressiveness. RWL magnitude potentially played an important role in maintaining the loss of muscle mass in athletes during the preparatory training phase (β –0.01 kg, *p* < 0.001). Therefore, an adequate regulatory programme should be integrated into the training plans of high-performance combat sports athletes to keep not only the athletes but also their coaches responsible for a proper weight control.

## 1. Introduction

The athletes involved in any of the Olympic combat sports branches such as judo, wrestling, boxing and taekwondo are categorised by body weight (BW) into weight classes seeking to mitigate the disparities in size and strength [1,2]. However, the majority of combat sport athletes take part in competitions of a weight category which is below their usual body weight [3] in order to attain the advantage over their weaker or smaller opponents [4,5,6,7,8]. Rapid Weight Loss (RWL) is a rapid reduction of weight over a brief time period in an attempt of attaining the norm meeting the requirements for a competition in a particular weight category. It is described as a temporary weight loss of up to 5% of a person’s weight over a short period of time (commonly within one week) [9]. Data obtained by many studies suggest that the prevalence of RWL practice among combat athletes ranged from 42% to 90% [5,10,11,12,13,14]. Before each competition, usually 2 to 3 days prior to the weigh-in procedure, combat sport athletes usually lowered their body weight by approximately 2% to 10% [15]. These practices appeared to be most common among athletes competing at higher contest levels where weight was managed by more aggressive strategies. Some most frequently deployed RWL techniques involve more intensive exercise; dehydration; use of sauna and rubber or plastic suits; reduction of energy intake; use of rubber or plastic suits for training; low intake of carbohydrates; limited consumption of fats; fasting; vomiting; diet pills, laxatives, and diuretics, all of which may have a negative impact on the performance of athletes or at least increase the risk of injury [16,17,18,19]. It was also well documented that RWL practice negatively impacts the health of athletes, leading to physical and psychological damage and may result in reduced bone density; worse performance of muscles; mood swings; dehydrated body condition; accelerated heart rate; poorer short-term memory, cognitive and mental function; or mounting anxiety, bad temper, weakness, depression, and a feeling of isolation [4,16,20]. Extreme cases of RWL have led to deaths as a result of dehydration and hyperthermia and myocardial infarction [21,22]. The threat of RWL has been recognised by sports bodies and position statements have been issued to reflect their stand [3,23]. In line with the criteria for prohibited methods stipulated in the World Anti-Doping Agency Code, Artioli et al. [15] even recommended to prohibit RWL practices in combat sports.

In summary, there are sufficient findings in scientific literature about the negative effects of the RWL magnitude not only on physical but also on mental health conditions among combat sports athletes. On the other hand, there is little evidence to generalise the impact of RWL magnitude on athletes’ body composition. To our knowledge, RWL decreases performance, bone mineral density, and muscle mass in both, males and females [24]. In addition, it was revealed that an acute restriction of food and fluid intake appeared to negatively affect fat-free mass and the indices of kidney function in combat sports athletes including a significant muscle damage induced by RWL [25,26]. However, up until now, the association between the RWL magnitude and the habitual weight status or body composition of elite combat athletes in the training process have not yet been evaluated.

Taking into account the international context of research, the available evidence on RWL among Lithuanian athletes in weight-sensitive sports is limited. Therefore, the requirement to identify the prevalence, magnitude and methods of RWL among athletes seems to be of primary importance. In the next step, this study aimed to identify the association between RWL and body composition among competitive combat athletes in Lithuania. We identified the following challenges: (1) the primary objective of this study was to investigate the prevalence, magnitude and self-reported methods of RWL; (2) the secondary objective of the study was to identify and evaluate the body composition in a sample of high-performance athletes; (3) the third objective of the study was to reveal the relationship between RWL and the individual weight components among combat sports athletes.

## 2. Materials and Methods

### 2.1. Participants and Procedures

In March and May 2018, an observational analytical non-experimental cross-sectional study was carried out. The target population of the survey was the elite combat sport athletes belonging to the Lithuanian Sports Centre (LSC) based in Vilnius, Lithuania, whose lists were approved by the Lithuanian National Olympic Committee (LNOC) of Lithuania. Our investigation included only the athletes with the history of attainment of an Olympic qualification quota place or those with a history of participation in the European Athletics Championships (ECh) and/or the World Athletics Championships (WCh) to obtain Olympic qualification. On the basis of the above mentioned inclusion criteria, during the preparatory training phase, 43 high-performance athletes of combat sports were selected and examined, who represented 84% of the candidates for the Lithuanian Olympic team. The pool of respondents included judo (*n* = 11), Greco-Roman wrestling (*n* = 19) and boxing (*n* = 13) athletes. The qualification standards the athletes had met before the study, served as major inclusion criteria.

### 2.2. Measures

#### 2.2.1. Anthropometric Measures

The athletes’ height was measured at the Lithuanian Sports Medicine Centre (LSMC) by means of a stadiometer (±0.01 m). The BW, the individual weight components (lean body mass (LBM) (in kg and %), muscle mass (MM) (in kg and %) and body fat (BF) (in kg and %) were measured at the LSC by means of the standing-posture 8-12-electrode multi-frequency bioelectrical impedance analysis (BIA) and using electrical signals of 5, 50, 250, 550 and 1000 kHz (X-SCAN body composition analyzer with the certification EN ISO (the European Union has adopted an international standard) 13,488; Kyungsan City, South Korea) [27,28]. The muscle and fat mass index (MFMI) of each athlete was determined by dividing the muscle mass (in kg) by body fat (in kg). Lean body mass, body fat, muscle mass and MFMI were assessed according to the norms identified for high-performance athletes (male and female) and validated by the scientists in Lithuania [27].

#### 2.2.2. Rapid Weight Loss Questionnaire

A questionnaire in line with the one deployed by Artioli et al. but adapted to the specifics of our study was used to explore the RWL practice [29]. The data were collected using a face-to-face questionnaire, translated to Lithuanian using back translation method. The Rapid Weight Loss Questionnaire (RWLQ) consisted of 18 items, and a special scoring system was used. The subjects with RWL practice records were assigned 3 points, while those without were assigned 0 points. When athletes reported the highest weight ever lost during their sporting career before the competition, 0.5 point was awarded for each kilogram of the weight lost. In addition, one point was awarded for 1 kg lost for those with the usual weight cut before the competition. The respondents were awarded scores of 5, 4, 3, 2, 1, and 0 after they had lost weight during the shortest period of time (from one to three days), and over increasingly longer periods of time, i.e., from 4 to 5 days, from 6 to 7 days, from 8 to 10 days, from 11 to 14 days, and from 14 days and longer, accordingly. One point was assigned per kilogram for the weight gained over one week after the competition. As for the methods deployed by athletes in losing weight, according to their responses such as “never”, “not anymore”, “almost never”, “sometimes”, and “frequently”, we awarded the scores 0, 0.5, 1, 2, and 3 accordingly. After calculating the total scores, it was obvious that higher scores corresponded to more aggressive weight loss utilising harmful methods and an increased risk of losing weight too rapidly. The self-reported history of the weight loss section highlighted the self-reported histories of athletes and was formed of three questions. Firstly, the athletes were enquired about the weight category they were competing in, secondly, if the athlete had moved from one weight category to another, thirdly, what weight class they belonged to over the previous off-season.

### 2.3. Statistical Analysis

The statistical analysis was performed using SPSS V.25 for Windows (Corporate headquarters 1 New Orchard Road, Armonk, NY, USA). Standard descriptive summary statistics were applied to characterize the responses. All the normally distributed continuous variables were presented as means ± standard deviations (SD), whereas the qualitative variables were presented as relative frequencies (in %). When normality was confirmed, independent and paired *t*-tests were used to assess the differences between some groups. Pearson correlations (parametric tests) were used to assess the relationship between the RWL score and the variables of interest (weight cut for competition (in kg) and weight regain in a week after competition (in kg)). 

The multinomial logistic regression analysis was used to relate the RWL scores and weight loss methods, later the results were presented by Odds Ratios (ORs) with 95% Confidence Interval (CI). As dependent variables must be categorical, continuous variables must be transformed, mostly via the classification of quartiles or percentiles. Thus, the dependent variable (RLWS (RWL score)) in this study was changed into the ordinal scale measurements by simply ranking the observations and using the values as a cut-off point of two subscales (20.7 ≥ RWLS > 20.7). The logistic model was adjusted for sports type, gender, and age. In this study, the parameters were estimated by means of the maximum likelihood method, also the appropriateness, adequacy and usefulness of the model were evaluated by the Wald (W) statistic, the estimated coefficient (β), with standard error (SE) (<5) and Nagelkerke R^2^ statistic. The multiple linear regression analysis was used to determine the association between the aggressive RWL methods (in score) and the individual weight components (MM (in kg) and BF (in kg)). The model was adjusted for sex, age and type of sport. The significance level *p* < 0.05 was determined for all statistical tests.

## 3. Results

### 3.1. Characteristics of the Subjects

The sample under analysis included 86% (*n* = 37) men and 14% (*n* = 6) women. The combat athletes’ age was within the range of 16 to 29. All the subjects participated in the National (Lithuanian) Championships and held prizes, 49% of combat athletes participated in the ECh (9% won medals), 26% of combat athletes participated in the WCh (12% won medals) and 5% of sportsmen participated in the Olympic Games (OG) (did not win medals).

The athletes’ training workload dimensions were totally consistent with the training plans that had been approved by the LSC and the LNOC. The Tokyo 2020 programme provided specifications of the training plans. Over the study period the athletes underwent testing which revealed their training period range of 7.4 ± 3.9 years, workouts performed 5.5 ± 0.9 days per week, an average duration of workout 147.5 ± 40.4 min per day.

Table 1 shows the distribution of combat athletes (in percentage) by the current weight class of participation in competitions in line with the weight classes being contested in judo, Greco-Roman wrestling and boxing qualification events for Tokyo 2020.

### 3.2. The Body Composition of Athletes

The body composition of combat athletes was assessed as indicated in Table 2. The BW, LBM and MM in subjects varied within the normal range. Nonetheless, the mean of LBM (83.9 ± 5.5%) in male athletes met the maximum (max.) limit (85%). ∆ LBM_in %_ (actual LBM_in %_—max. recommended LBM_in %_) was—1.1 ± 0.8% (95% PI: −0.9–0.7). The LBM (78 ± 2.7%) of female athletes was not different from the max. limit (80%). ∆ LBM_in %_ (actual LBM_(in %)_—max. recommended LBM_in %_) was—2 ± 1.1% (95% PI: −4.8–0.9).

The mean of BF in male athletes involved in sports such as judo, Greco-Roman wrestling and boxing (16 ± 5.4% of BW) was acceptable and met the average (avg.) recommended limit (17% of BW). ∆ BF_in %_ (actual BF_in %_—avg. recommended BF_in %_) was −1 ± 0.9% (95% PI: −2.8–0.8). Meanwhile, BF of female athletes involved in judo was 22.1 ± 2.6% of body weight, which was acceptable (20–24% of BW) and the BF was not different from the avg. recommended rate (22%): ∆ BF_in %_ (actual BF_in %_—avg. recommended BF_in %_) was 0.1 ± 1.1% (95% PI: −2.6–2.8). Apart from that, the female athletes’ MFMI (3.4 ± 0.6) was consistent with the avg. one (3–3.9) (∆ MFMI (actual MFMI—minimum recommended MFMI) was 0.4 ± 0.2; 95% PI: −0.2–2.9). MFMI in male athletes was high and there was no statistically significant difference from the max. range of 4.7 to 6. The paired sample *t*-test between the MFMI in male athletes (5.7 ± 2.8) and the max. MFMI limit were found without statistical significance (∆ MFMI (actual MFMI—max. recommended MFMI) was −0.3 ± 0.4; 95% PI: −1.2–0.6).

### 3.3. Prevalence, Magnitude and Methods of RWL 

**Figure 1:**
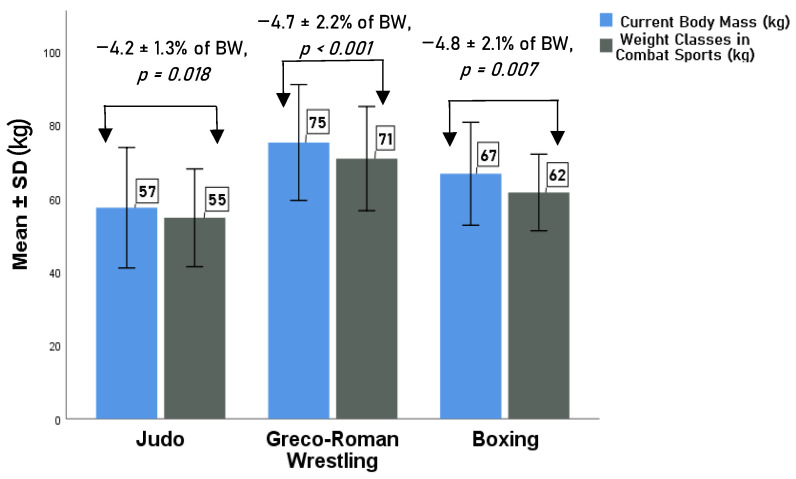
Prevalence of RWL (in kg and % of BW) among elite combat sports athletes (*t*-test was used).

The prevalence of the reported RWL among elite combat sports athletes was 88%, which means that 38 out of 43 interviewed subjects reported practicing RWL (Figure 1). 

Table 3 presents the profile of sport practice and weight loss history reported by the combat sport athletes. On average, the athletes began participating in combat sports before turning 10 years old and had been participating competitively after they reached the age of 11 years. In addition, the athletes began to resort to weight-cutting as early as 9 years old, with the mean age of 12.8 ± 2.1 years. The athletes were engaged in weight loss practices that took a short duration of time (4.9 ± 3.3 days) and they had 7.1 ± 5.6 episodes of weight reductions in the previous year. Their current mean weight (68.1± 16.7 kg) was almost similar to the previous off-season weight (67.8 ± 16.7 kg). However, the average ever lost most weight (3.1 ± 1.5 kg) was less than 4 kg, accounting for almost 5% of their body weight, and 104.1 ± 28.3 kg of this weight loss most frequently recovered a week later. 

In this sample of combat athletes, the RWLS was 21.6 ± 4.6. Multivariate logistic regression was constructed to obtain how the predictors such as different self-reported RWL methods (skipping one or two meals, restricting fluids, increased exercise, training with rubber/plastic suits) may predict the magnitude of RWL (RWLS ≥ 20.7). ORs of covariates in multivariate logistic regression model were adjusted for the sports type, sex, and age. The results of multivariate analysis were displayed in Table 4. The regression of model identified that the athletes at high risk of RWL were significantly more likely to skip one or two meals (AOR 6.3; 95% CI: 1.3–31.8), to increase exercise (AOR 3.6; 95% CI: 1.0–12.5) and to train with rubber/plastic suits (AOR 3.2; 95% CI: 0.9–11.3). Even though the use of energy restriction strategies such as skipping one or two meals is a common practice, the methods to diminish body water stores (i.e., restrict fluid intake) were also frequently practised for RWL by this cohort (AOR 5.5; 95% CI: 1.0–31.8). In all, 92.1% of the athletes used fluid restriction “sometimes” or “always”, and 68.4% of them practised this method between 1 and 24 h before weigh-ins. More specifically, 68.4% of the weight-reducing athletes drank less than 500 mL of drinks or water on the last day before the competition, while 23.7% of the athletes sustained from drinking fluids at all. According to our study, the athletes experienced dehydration due to the reduced fluid intake which corresponded to 1.4 ± 0.7% of BW. A more detailed analysis of the frequency of the weight loss methods deployed by the combat sports representatives is given in Table S1.

According to our study, a correlation has been found between the RWLS and the weight cut/lost for the competition (r = 0.39, *p* = 0.016), and the weight regained in a week after the competition (r = 0.35, *p* = 0.033) in a sample of combat athletes.

### 3.4. The Association between RWL and Body Composition

Multivariate liner regressions were constructed to obtain how the predictor RWL magnitude may predict the body composition (MM (in kg), BF (in kg), MFMI) of combat sports athletes. Multivariate linear regression model was adjusted for athlete sport, sex and age. The results of multivariate analysis were displayed in Table 5. After using a multivariate linear regression method, we found that with a 95% confidence level the MM decreased from 0 to −0.03 kg depending on increased RWLS for 1 point (β −0.01, *p* < 0.001). Meanwhile, no association has been established between the current MM status (in kg), MFMI and the application of more aggressive RWL methods (in score) (Table 5).

## 4. Discussion

### 4.1. The Prevalence of RWL 

This study has been an initial effort to explore the scope of prevalence of the RWL practices in the Lithuanian elite combat athletes which revealed the real picture of a rapid weight loss practice observed in 88% of the subjects (73% judokas, 95% Greco-Roman wrestlers, and 92% boxing Olympic athletes) over the previous year. This figure stood at a similar level in comparison to that observed for athletes practising similar combat sports such as wrestling (42–90%) [11,14,18], judo (63–90%) [12,16,30] and boxing (50–100%) [1], and it showed the prevalence of the ongoing practice of RWL among combat sports representatives, without regard to the sport branch.

Additionally, according to our study, the athletes began to adjust their bodies to weight-cutting being as young as 9 years of age, with the mean age of 12.8 ± 2.1. Similar results were highlighted in scientific literature with evidence on about 60% of judo athletes starting weight cycling before fights at the age of 12–15 and wrestlers at the age of 15.5 ± 2.4 [1,5,12]. It was also shown that the normal growth and development of adolescents were interrupted due to a disturbance in hormone levels induced by continuous by RWL practices [11,15,31,32,33]. 

### 4.2. The Magnitude of RWL

Combat sports athletes most commonly lose ≥ 5% of BW over the seven days prior to weigh-in [1]. According to our data, the average BW loss found before the competition in a sample of the Lithuanian combat athletes varied within the range of 3.1 ± 1.5 kg (4.6 ± 2.0% of BW). It should be taken into consideration that under some conditions, it is allowed to reach from 5% to 8% of BW loss with an acceptable slight effect on the health condition and performance of athletes [34,35]. As the National Collegiate Athletic Association (NCAA) weight loss guidelines stipulate, the Minimum Wrestling Weight (MWW) is grounded on 5% of BF as the lowest BF percentage allowed and the levels of safe weight loss attainable before the first weigh-in procedure at the competition— namely, losing no more than 1.5% of BW per week [36]. In line with the data from our study, male athletes involved in sports such as judo, Greco-Roman wrestling and boxing had a high BF percentage (16 ± 5.4%). Therefore, before competing, the BW of combat athletes can be adjusted as much as possible. On the other hand, the athletes’ LBM was relatively high and led to a high ratio between muscle mass and fat mass (5.7 ± 2.8). Therefore, BW loss should not be a priority for elite athletes in the run-up to the competition.

### 4.3. The Methods of RWL 

According to the scholarly literature, the most frequently practised methods to start RWL were fluid restriction [5,6,20,25,37,38,39,40,41,42,43,44], energy restriction [25,37,40,45], wearing rubber/plastic suits [5,37,39,41], increased exercise [37,40,42], heated room training [37,39], use of sauna [37,41], spitting [5], using laxatives [41] and gradual dieting [37]. However, according to our study, aggressive RWL methods among the Lithuanian combat athletes were less common. Additionally, emphasis can be laid on the fact that in a sample of Lithuanian athletes, those at high risk of RWL were significantly more likely to skip one or two meals, to increase exercising and to train with rubber/plastic suits. 

On the other hand, the results obtained from our study were in line with the findings of other studies carried out by previous researchers and showed that among the most prevailing methods employed by the cohort of combat athletes in Lithuania for RWL was the method of reducing the body water stores (i.e., fluid restriction). The human body of the general population is composed of ~60% of water and most probably larger amount in athletes with higher LBM levels [46]. Taking into account the size of the body and the short time frame for the occurrence of the possible fluctuations, it is not surprising that dehydration appeared to be the primary RWL strategy practiced by a number of combat sports representatives. According to our study, fluid restriction was used by 92.1% of the combat athletes in Lithuania, while 68.4% of weight-reducing athletes consumed less than 500 mL of drinks or water on the last day before the competition, while 23.7% of athletes did not consume any fluids at all. Therefore, the athletes experienced dehydration as a result of reduced fluid intake which corresponded to 1.4 ± 0.7% of BW. On the one hand, mild dehydration (<2% BW) tends not to affect the performance, while on the other hand, a larger scope may pose problems, especially during the limited time to restore the rehydrated post weigh-in [34,35]. In other words, the athletes can take a choice from two methods available to decrease their bodily water, either to take in a lower amount of fluids and/or excrete a larger amount of fluids. The restriction in fluid intake that lasts for 24 h (<300 mL) may result in 1.5–2.0% BW loss [47]; however, the athletes most frequently achieve a loss larger than this as a result of the accelerated sweating rate via either active (as a result of intensive exercise) and/or passive techniques deployed (i.e., sauna rooms, heated environments, the use of sweat suits, etc.) [1,7]. This data was in line with the data obtained from our study showing that elite combat athletes from Lithuania were combining not only an extremely low fluid intake, but were also exercising more intensively and training with rubber/plastic suits. Therefore, future studies should focus and verify more accurate levels of dehydration observed in athletes. In addition, it should be highlighted that severe (or even moderate) dehydration employed as a tool to lose weight in weight controlled sports accelerates the risk of acute cardiovascular ailments [15,48]. The athletes who use thermal exposure seeking to achieve such dehydration run an increased risk suffering from heat stroke or heat illness [15]. Additionally, acute dehydration may significantly impact the electrolyte concentration composition which could impact the cell fluid balance, metabolism and result in the impairment of the neuromuscular function [49,50]. Scholarly works suggest that in-depth dehydration is also likely to induce alterations in the morphology of the brain and pose the risk of potential brain injuries as a consequence of head traumas triggered by strikes [51,52].

What is more, typically in a wide range of sports, prior to competitions, the nutritional status includes “fine tuning” procedures consistent to satisfying the needs of comfort, repetitive and habitual practices rather than clinging to excessive weight adjusting patterns. Even though there is insufficient evidence of research into the specific dietary patterns the athletes select to achieve RWL [53], the most frequently observed acute caloric restriction tends to impact the performance through the depleted glycogen depots [35]. Our study showed that energy restriction strategy (skip one or two meals) was frequently used by cohort of combat sports athletes. Therefore, this strategy of energy restriction had the association with the magnitude of RWL. The authors also point out that earlier research carried out by them in Lithuania found that the wrestlers during the RWL period followed low-energy and low-carbohydrate diet [54]. More specifically, low total energy intake contributed to the insufficiency of the intake of vitamins A, B_1_, B_2_, PP, D, E, B_6_, folic acid, and the intakes of minerals such as potassium, calcium, phosphorus, magnesium, iron, zinc during the rapid bodyweight reduction. Greenhaff et al. [55] claimed that ‘a diet low in carbohydrates even when the food consumption is sufficient, could impact the buffering properties of the blood‘. The interacting effect of RWL and the muscle hydrogen ion efflux could be regulated down by a low carbohydrate intake and can result in increased fatigue during the intense muscular contractions [45].

### 4.4. The Body Composition and RWL 

The combat athletes were engaged in RWL practices taking a short period of time and had 7 episodes of weight reductions in the previous year. The average most weight ever lost accounted for an almost 5% of their body weight, and 104% of this weight loss was usually regained in the week after a fight. Additionally, we found the correlation between RWLS and the weight cut for the competition, and the weight regained in a week after the competition in a sample of combat athletes. It can be explained that applying more aggressive RWL methods before the competition relates to how much weight combat athletes restore after the competition. This type of weight cycling may lead to MM damage and catabolism [24,25,26]. In this context the results of our study were interesting as the relationship was found between RWL and body composition. Utilising more aggressive and harmful RWL methods related to decreased MM during the preparatory training phase in a sample of Lithuanian combat sports athletes. Further research will be necessary to identify the effect of RWL magnitude on the athlete body composition in the relation to manipulations with the athlete‘s body mass.

### 4.5. Limitations of the Study

Our study had a few limitations. The study was carried out by a single centre and the size of the sample was small. A larger sample and an international multi-centre size could represent the findings of the quantitative data more accurately. In addition, the survey tools were unable to determine and provide a cut-off to indicate where the weight management behaviour was dangerous and could potentially cause harm. In the view of the above, scholars have now developed a new and safe (in terms of hyponatremia) method of ‘water loading’ that promotes dehydration [56,57,58,59,60]. In connection with this new RWL method, the limitation of our study was the absence of additional questions on the mentioned method in the questionnaire (RWLQ) we used [29]. Moreover, some findings were based on the data that was self-reported retrospectively; thus, some of the biased responses on behalf of the athletes were unavoidable. 

## 5. Conclusions

This study proves high levels of RWL practices in the Lithuanian athletes of elite combat sports (88%) five days prior to the competition, resulting in the reduction of the average of 3.1 kg (4.6%) of their body weight. The athletes began to resort to weight-cutting as early as 9 years old, with a mean age of 12.8 ± 2.1 years. The combination of practiced weight loss techniques such as skipping one or two meals, restricting fluids, increased exercise, training with rubber/plastic suits may predict the magnitude of RWL. Utilising more aggressive and harmful RWL methods before the competition is associated to on how much weight combat athletes are cutting for the competition and regaining after the event. RWL being a widely spread practice among athletes of combat sports, can trigger negative consequences of the athletes’ body composition. RWL magnitude potentially plays an important role in maintaining the loss of muscle mass during the preparatory training phase. Therefore, an adequate regulatory programme should be integrated into the training plans for high-performance combat sports athletes to keep not only the athletes but also their coaches responsible for a proper weight control.

## Figures and Tables

**Table 1:** Weight classes of combat sports athletes.

Sport	Male		Female		Weigh-In Procedures
	Weight Classes	% of Athletes	Weight Classes	% of Athletes	
Judo	<60 kg	80.0	<48 kg	66.7	Have a trial 1 h weight-in period the day before the competition in the evening, and the next day during the checkweigher.
	66 kg	20.0	52 kg	33.3	
	73 kg	-	57 kg	-	
	81 kg	-	63 kg	-	
	90 kg	-	70 kg	-	
	100 kg	-	78 kg	-	
	+100 kg	-	+78 kg	-	
Greco-Roman Wrestling	<60 kg	5.3	-	-	The weigh-in for each category always takes place on the day before the beginning of the competition concerned and lasts 30 min.
	67 kg	36.8	-	-	
	77 kg	36.8	-	-	
	87 kg	10.5	-	-	
	97 kg	5.3	-	-	
	130 kg	5.3	-	-	
Boxing	Flyweight (<52 kg)	15.4	48–51 kg	-	There is a weight-in of all competitors every morning throughout the competition (several days) and must keep their weight class limit.
	Featherweight (57 kg)	30.8	54–57 kg	-	
	Lightweight (63 kg)	23.1	57–60 kg	-	
	Welterweight (69 kg)	7.7	64–69 kg	-	
	Middleweight (75 kg)	7.7	69–75 kg	-	
	Light heavyweight (81 kg)	15.4	-	-	
	Heavyweight (81–91 kg)	-	-	-	
	Super heavyweight (91+ kg)	-	-	-	

**Table 2:** Body composition of combat sports athletes.

Variables	Judo		Greco-Roman Wrestling	Boxing	Normative Male/Female
	Male	Female	Male	Male	
Height (m)	1.63 ± 0.17	1.64 ± 0.07	1.77 ± 0.08	1.73 ± 0.08	
BW (kg)	59.4 ± 25.7	55.8 ± 2.3	75.1 ± 15.7	66.5 ± 14.2	
LBM (in kg)	48.7 ± 14.5	43.6 ± 2.8	62.3 ± 11.2	55.4 ± 7.7	
LBM (% of BW)	85.1 ± 9.0	78.0 ± 2.7	83.1 ± 3.8	84.3 ± 6.2	75–85/70–80
MM (in kg)	45.3 ± 13.1	40.4 ± 2.7	57.7 ± 10.1	51.6 ± 6.7	
MM (% of BW)	79.3 ± 8.9	72.2 ± 2.7	76.5 ± 4.6	78.7 ± 6.2	74–80/64–80
BF (in kg)	10.7 ± 11.5	12.2 ± 1.4	13.2 ± 5.3	11.2 ± 6.7	
BF (% of BW)	14.9 ± 9.0	22.1 ± 2.6	16.7 ± 3.9	15.7 ± 6.2	15–19 ^1^/20–24 ^2^
MFMI	6.9 ± 3.5	3.4 ± 0.6	4.8 ± 1.4	6.4 ± 3.9	4.7–6 ^1^/3–3.99 ^3^

^1^—a large (acceptable) BF (% of BW); ^2^—an average (optimal) BF (% of BW); ^3^—an average MFMI; The data are presented as means ± standard deviation (SD). BW–body weight; LBM–lean body mass; MM–muscle mass; BF–body fat; MFMI–muscle and fat mass index.

**Table 3:** Profile of sport practice and weight loss history reported by the combat sports athletes.

Profile of Sport Practice and Weight Loss History	Judo	Greco-Roman Wrestling	Boxing	Total
Prevalence of the reported RWL (%)	73	95	92	88
Total RWLS	20.3 ± 6.2	20.9 ± 3.6	23.5 ± 4.8	21.6 ± 4.6
Age at the start of sport practice (years)	8.7 ± 3.1	9.8 ± 2.2	10.9 ± 2.9	9.9 ± 2.7
Age at the start of competition (years)	9.2 ± 2.4	10.9 ± 2.1	10.2 ± 4.9	10.4 ± 4.7
Fights over previous 12 months	10.4 ± 4.2	15.8 ± 9.5	13.3 ± 5.2	13.7 ± 7.4
Age at the start of weight cut (years)	12.5 ± 1.1	12.9 ± 2.5	13.2 ± 1.9	12.8 ± 2.1
Off-season weight (kg)	57.1 ± 16	75.3 ± 15.8	66.3 ± 13.7	67.9 ± 16.8
Frequency of weight reductions in previous year	5.6 ± 3	8.2 ± 7.3	6.5 ± 3.7	7.1 ± 5.6
Duration of weight reduction (days)	5.3 ± 4	4.7 ± 3.3	4.9 ± 2.8	4.9 ± 3.3
BW regain in a week after fight (kg)	2.6 ± 0.5	3.4 ± 1.6	2.8 ± 1.5	3.1 ± 1.4
BW regain in a week after fight (% of weight cut)	117.7 ± 23.3	106.7 ± 30.6	91.4 ± 24.2	104.1 ± 28.3

BW—body weight; RLWS—rapid weight loss score. The data are presented as means ± standard deviation (SD).

**Table 4:** The association between the RWL methods deployed by the combat sports representatives and the RWL score.

RWL Score ^a^ (Score > 20.7)	β	SE	W	*p*	AOR (95% CI)
Skipping one or two meals	1.8	0.8	5.0	*0.025*	6.3 (1.3, 31.8)
Restricting fluids	1.7	0.9	3.6	*0.05*	5.5 (1.0, 31.8)
Increased exercise	1.3	0.6	4.1	*0.043*	3.6 (1.0, 12.5)
Training with rubber/plastic suits	1.2	0.6	3.3	*0.049*	3.2 (0.9, 11.3)
Constant	−10.4	3.4	9.2	*0.002*	0

^a^—reference category is RWL score (score ≤ 20.7); β—the estimated coefficient; SE—the standard error (SE) of β; W—the Wald statistic; OR—Odds Ratio; CI—confidence interval; Nagelkerke R^2^ = 0.66; ORs in the logistic model was adjusted for the sports type, sex and age (AORs).

**Table 5:** The association between MM, BF, MFMI and RWL score.

RWL (Score)	β	95% CI	*p*
MM (kg)	−0.01	(−0.03; 0)	*<0.001*
BF (kg)	0.06	(0.04; 0,1)	*0.087*
MFMI	0.08	(0.03; 0.1)	*0.121*

The association between MM, BF, MFMI and RWL score was estimated controlling for athlete sport, sex, and age (adjusted for sports type, sex and age). F (6, 36) = 7.2, *p* < 0.0001, R^2^ = 0.56. MM—muscle mass; BF—body fat; MFMI–muscle and fat mass index.

## Data Availability

Data are available on request.

## Supplementary Materials

The following supporting information can be downloaded at: https://www.mdpi.com/article/10.3390/healthcare10040665/s1, Table S1: Frequency analysis of the weight loss methods reported by the combat sport athletes; Table S2: Acronyms and terminology.
