# Supplementary material for: The Association between Rapid Weight Loss and Body Composition in Elite Combat Sports Athletes
Source: Healthcare (Basel). 2022 Apr 1;10(4):665. doi: 10.3390/healthcare10040665 (PMC9031560; doi:10.3390/healthcare10040665)
Supplement: Supplementary file 1 [file healthcare-10-00665-s001.zip › healthcare-1637951-supplementary.pdf]

SUPPLEMENTARY: TABLES

Table S1. Frequency analysis (in percentage) of the weight loss methods reported by the combat sport athletes.

Method	Never	Do not use anymore	Almost never	Sometimes	Always
Gradual dieting	52.6	31.6	5.3	7.9	2.6
Skipping one or two meals	7.9	10.5	26.3	18.4	36.8
Fasting	21.1	21.1	10.5	28.9	18.4
Restricting fluids	5.3	7.9	13.2	42.1	31.6
Increased exercise	21.1	15.8	23.7	18.4	21.1
Heated training rooms	23.7	7.9	23.7	21.1	23.7
Sauna	15.8	15.8	28.9	18.4	21.1
Training with rubber/plastic suits	39.5	7.9	21.1	13.2	18.4
Wearing winter suit all day	73.7	10.5	7.9	5.3	2.6
Spitting	65.8	10.5	18.4	5.3	0
Laxatives	100	0	0	0	0
Diuretics	92.1	2.6	5.3	0	0
Diet pills	100	0	0	0	0
Vomiting	92.1	7.9	0	0	0

Table S2. Acronyms and terminology.

Term	Definition
Δ	Delta
AOR	Adjusted Odd Ratio
Avg.	Average
BIA	Bioelectrical Impedance Analysis
BF	Body Fat
BW	Body Weight
CI	Confidence Interval
ECh	European Athletics Championships
LBM	Lean Body Mass
LNOC	Lithuanian National Olympic Committee
LSC	Lithuanian Sports Centre
LSMC	Lithuanian Sports Medicine Centre
Max.	Maximum
MFMI	Muscle and Fat Mass Index
MM	Muscle Mass
RWL	Rapid Weight Loss
RWLS	Rapid Weight Loss Score
RWLQ	Rapid Weight Loss Questionnaire
SD	Standard Deviation
SE	Standard Error
WCh	World Athletics Championships
